# Burden of fecal colonization with extended-spectrum beta-lactamase producing enterobacteriaceae among food handlers in Africa: A systematic review and meta-analysis

**DOI:** 10.1371/journal.pone.0340571

**Published:** 2026-08-24

**Authors:** Amanuale Zayede, Yitayih Wondimeneh, Sirak Biset, Mitkie Tigabie, Henok Worku, Biruktawit Abebe, Haymanot Tilahun, Eshet Gebrie, Mucheye Gizachew

**Affiliations:** 1 Department of Medical Microbiology, School of Biomedical and Laboratory Sciences, College of Medicine and Health Sciences, University of Gondar, Gondar, Ethiopia; 2 Department of Medical Laboratory Sciences, College of Health Science, Woldia University, Woldia, Ethiopia; 3 Department of Medical Laboratory Sciences, College of Medicine and Health Sciences, Injibara University, Injibara, Ethiopia; 4 Department of Clinical Chemistry, School of Biomedical and Laboratory Sciences, College of Medicine and Health Sciences, University of Gondar, Gondar, Ethiopia; 5 Department of Medical Laboratory Sciences, College of Health Sciences, Debark University, Debark, Ethiopia; Chukwuemeka Odumegwu Ojukwu University, NIGERIA

## Abstract

**Background:**

Antimicrobial resistance caused by extended-spectrum beta-lactamase–producing Enterobacteriaceae (ESBL-PE) has emerged as a major threat to global public health. The human gastrointestinal tract represents a critical ecological niche for these organisms, enabling silent carriage and onward transmission. Food handlers are of particular concern because they can facilitate indirect transmission of resistant bacteria through food preparation and handling. Despite the availability of primary studies, evidence focusing specifically on food handlers in Africa remains fragmented. This systematic review and meta-analysis therefore aimed to estimate the pooled prevalence of fecal colonization with ESBL-PE among food handlers in Africa.

**Methods:**

This review was conducted according to a protocol registered in PROSPERO (ID: CRD420251075141). A systematic search was performed from September 5–15, 2025 in PubMed, Google Scholar, and Hinari/Research4Life to identify relevant studies. The methodological quality of the included studies was appraised using the Joanna Briggs Institute (JBI) critical appraisal tool. Data were extracted into Microsoft Excel 2019 and analyzed with Stata software version 17. Given significant heterogeneity among the studies (I^2^ = 98.3%, *p* < 0.001), a random-effects model (DerSimonian and Laird) was used to calculate the pooled prevalence. To investigate the substantial heterogeneity, we performed subgroup and sensitivity analysis. The potential for publication bias was assessed using funnel plot and Egger’s test.

**Results:**

The meta-analysis incorporated nine studies with publication year ranged between 2012 and 2023, involving 4,061 participants. The pooled fecal colonization rate of ESBL-PE among food handlers in Africa was 23.64% (95% CI: 15.3, 31.94%). A high degree of heterogeneity was observed (I^2^ = 98.3%, *p* < 0.001). The most prevalent ESBL-PE species identified was *E. coli*, with a pooled prevalence of 85.83% (95% CI: 79.97, 91.69%, I^2^ = 95.56%, *p* < 0.001), followed by *Klebsiella* species at 23.92% (95% CI: 18.48, 29.35%, I^2^ = 0.00%, *p* = 0.65).

**Conclusion and recommendations:**

This meta-analysis demonstrates that approximately one in four food handlers were identified as ESBL-PE carriers in Africa based on pooled estimates. These findings highlight the need for strengthened food safety regulations, routine screening of high-risk occupational groups, and enhanced antimicrobial stewardship programs to limit the spread of resistant Enterobacteriaceae.

## Introduction

Enterobacteriaceae comprise a diverse group of Gram-negative bacteria that commonly inhabit the intestinal tract of humans and animals, while also causing a wide range of community- and healthcare-associated infections [1,2]. Of particular concern are ESBL-producing Enterobacteriaceae (ESBL-PE), which have been classified by the World Health Organization (WHO) as critical-priority pathogens due to their resistance profile and public health impact [3]. Species such as *Escherichia coli* and *Klebsiella pneumoniae* are among the most frequently identified ESBL producers and are responsible for severe infections worldwide [4]. The Infectious Diseases Society of America (IDSA) has identified them as two of the six pathogens for which new drugs are urgently required to address the issue of resistance development [5]. According to the Global antimicrobial resistance (AMR) and Use Surveillance System (GLASS) Report 2022, resistance rates to meropenem and third-generation cephalosporins in E. coli infections increased by more than 15% in 2020 compared to 2017 [6].

Experts predict that by 2050, AMR could cause approximately 1.91 million deaths annually worldwide. Without effective interventions, cumulative AMR-related deaths may reach 39.1 million between 2025 and 2050, demanding urgent global action to mitigate this crisis [7]. Intestinal colonization with ESBL-PE is often asymptomatic; however, colonized individuals remain at increased risk of subsequent invasive infections and may unknowingly transmit resistant organisms to others [8].

The production of beta-lactamases, enzymes that hydrolyze and inactivate beta-lactam antibiotics, is one of the major mechanisms of AMR in bacteria. The ESBL are a particularly dangerous subset of these enzymes that can break down third-generation cephalosporins (e.g., cefotaxime, ceftriaxone, ceftazidime) and monobactams (e.g., aztreonam) [4,9]. This resistance is acquired by plasmid-mediated mutation encoding for the parent enzymes, either by amino acid substitution in the active site such as the case for Temoneira (TEM) or sulfhydryl reagent variable (SHV; class A) enzymes or by inter bacteria gene transfer like the case of Cefotaxime-Munich (CTX-M) [10,11].

According to the Ambler molecular classification, ESBLs predominantly fall into Class A and Class D serine β-lactamases. Class A encompasses commonly encountered plasmid-borne enzymes such as TEM, SHV, and CTX-M, all of which are susceptible to inhibition by clavulanate. In contrast, Class D comprises oxacillinases (OXA-type enzymes), which are typically resistant to clavulanate and exhibit potent hydrolytic activity against oxacillin [12]. Under the Bush‑Jacoby functional scheme, ESBLs are placed within Group 2be, which includes serine β‑lactamases that are inhibited by clavulanic acid. Additionally, certain OXA‑type ESBLs are assigned to Group 2d, a group characterized by clavulanate‑resistant enzymes [13].

The ESBL-PE are among the most urgent AMR threats, as identified by the United states (US) Centers for Disease Control and Prevention (CDC) [14]. These pathogens severely limit treatment options, as they often require the last resort antibiotics such as Carbapenems [15].

The spread of these resistant bacteria leads to higher mortality rates, prolonged hospital stays, and increased healthcare costs [16,17]. The transmission of ESBL-PE occurs through multiple reservoirs, including colonized patients, contaminated medical equipment and food products [18]. Additionally, the gut microbiota can serve as a reservoir for resistance genes, facilitating their transfer to pathogenic strains [19].

Previous systematic reviews have documented high rates of fecal colonization with ESBL-PE among hospitalized patients and community populations in Africa [20–24]. Nevertheless, food handlers represent a distinct occupational group with unique exposure pathways, including frequent contact with raw poultry and meat, inadequate workplace food‑safety enforcement, and limited access to hygiene infrastructure [25]. Despite their potential role in disseminating AMR bacteria through the food chain, evidence on ESBL-PE colonization among food handlers in Africa has not been comprehensively synthesized. To address this gap, the present systematic review and meta-analysis aimed to determine the pooled prevalence of fecal colonization with ESBL-PE among food handlers across African countries.

## Materials and methods

### Study design and protocol registration

This systematic review and meta-analysis was conducted in accordance with the Preferred Reporting Items for Systematic Reviews and Meta-Analyses (PRISMA) 2020 guidelines **(****S1 Table**) [26]**.** The study protocol was prospectively registered in the International Prospective Register of Systematic Reviews (PROSPERO) under the registration number CRD420251075141**.**

### Literature search strategy

A comprehensive literature search was performed between September 5 and September 15, 2025 using PubMed, Google Scholar, and Hinari/Research4Life databases. To ensure completeness, the reference lists of all eligible articles were also manually screened to identify additional relevant studies, including gray literature and unpublished reports.

The search strategy combined relevant Medical Subject Headings (MeSH) terms and free-text keywords related to fecal colonization, ESBL-PE and food handlers. Boolean operators (“AND” and “OR”) were applied to refine the search.

An example of the full search string used for PubMed is provided for transparency: ((“Fecal carriage” OR “Fecal colonization” OR “Gut carriage” OR “Gut colonization” OR “Intestinal colonization” OR “Intestinal carriage” OR Colonization OR Carriage)) AND ((“Extended spectrum beta lactamase” OR “Extended spectrum β lactamase” OR ESBL OR ESβL OR Beta-Lactamase)) AND ((Enterobacteriaceae OR Enterobacterales OR “Gram-Negative Bacteria” OR “Gram-Negative Bacteria”[Mesh] OR Enterobacteriaceae[Mesh] OR Klebsiella OR Klebsiella[Mesh] OR “Escherichia coli” OR “Escherichia coli”[Mesh] OR E.coli OR Shigella OR Shigella[Mesh] OR Salmonella OR Salmonella[Mesh])) AND ((Africa OR Africa[Mesh] OR “Sub Saharan Africa” OR “Africa South of the Sahara”[Mesh])) **(****S2 Table****)**.

### Eligibility criteria

Studies were selected using the CoCoPop (Condition, Context, and Population) framework [27], where the condition was fecal colonization with ESBL-PE, the population was food handlers, and the context was African countries.

#### Inclusion criteria.

We included observational studies (cross-sectional, case-control, and cohort designs) that investigated the prevalence or factors associated with laboratory confirmed ESBL-PE carriage. The population of interest was adult food handlers (≥18 years) employed in any food service setting, including restaurants, street vending, and institutional catering services across any African country. Eligible studies had to confirm ESBL-PE status through laboratory analysis of stool or rectal swab samples. There were no restrictions on the publication date. To reduce publication bias, gray literature and unpublished studies from preprint servers and conference abstracts were also considered.

#### Exclusion criteria.

Reviews, case reports, case series, animal studies, and studies not based on stool samples were excluded. Research focusing on food handlers with acute diarrheal illness at sampling was also excluded to avoid capturing transient colonization.

### Study selection

Study selection followed the PRISMA 2020 guidelines (**S1 Table**) [26]. The process of study identification, screening, eligibility assessment, and final inclusion is illustrated in the PRISMA flow diagram. All records identified through the database searches were imported into EndNote version 9.2 for reference management, and duplicate were removed. The study selection process was carried out in two phases using the predefined CoCoPop framework [27]. First, two reviewers (AZ. and MG.) independently screened the titles and abstracts of all identified citations against the eligibility criteria. Second, the full texts of the potentially relevant studies were retrieved and assessed independently by the same reviewers for final inclusion. Any disagreements between the reviewers at either stage were resolved through discussion or by consulting a third and fourth reviewers (YW. and SB.) [28].

**Fig 1:**
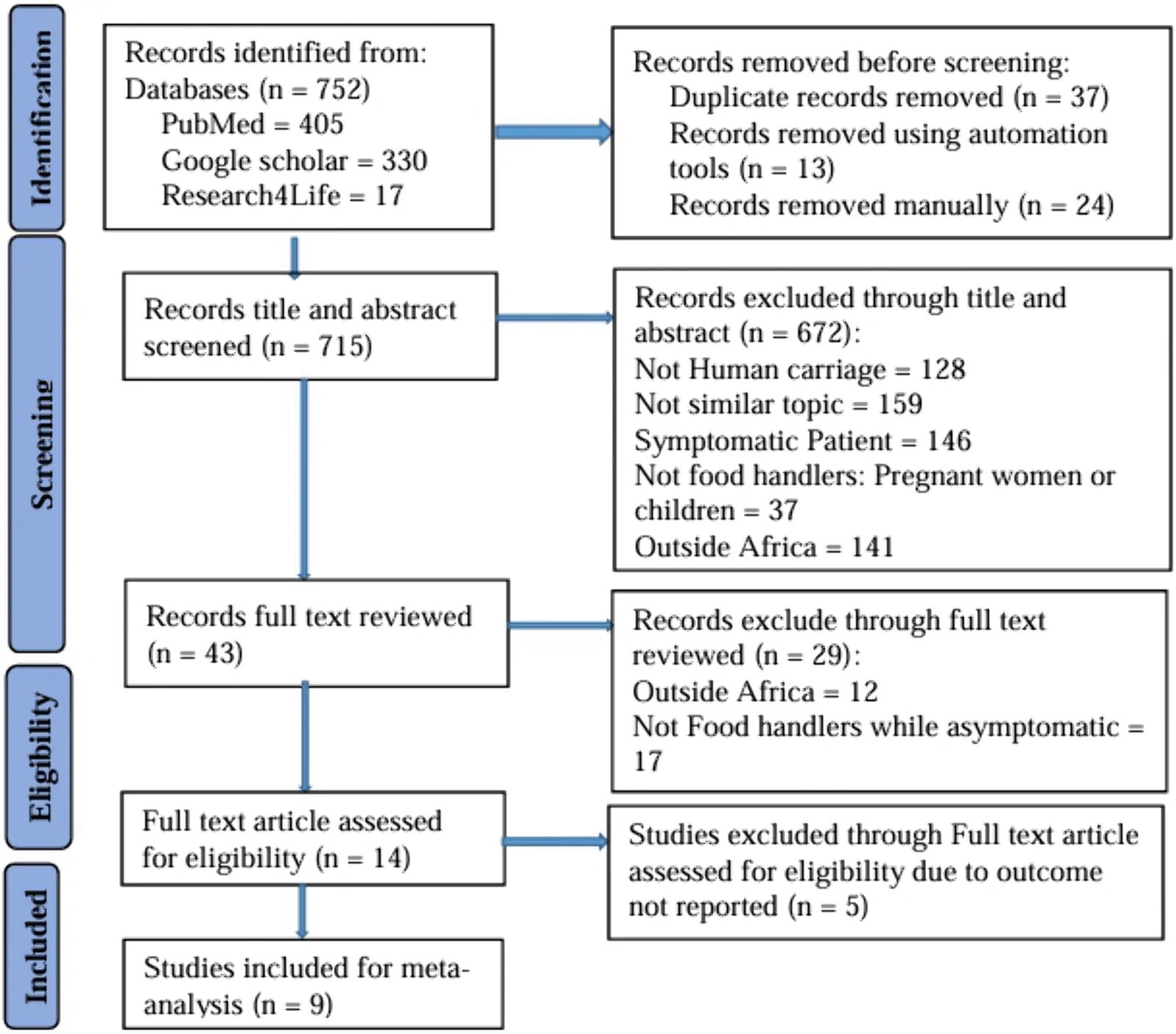
PRISMA 2020 flow diagram illustrating the study selection process for the systematic review and meta-analysis.

### Quality appraisal

The methodological quality of the included studies was evaluated using the Joanna Briggs Institute (JBI) critical appraisal checklist for prevalence studies [27]. Two reviewers (AZ. and MG.) independently applied the relevant JBI checklist to each study. These checklists contain nine items, and studies were scored as follows: 0–4 (low quality), 5–7 (moderate quality), and 8–9 (high quality) [29]. Only studies achieving a score of 5 or higher (i.e., moderate or high quality) were incorporated into the final analysis. Any discrepancies between the reviewers’ assessments were resolved through discussion or by consulting a third and fourth reviewers (YW. and SB.) [28].

### Outcome variable measurement

The primary outcome for this meta-analysis was the pooled prevalence of fecal colonization with ESBL-PE among adult food handlers across Africa. For each included study, the prevalence was calculated as the proportion of tested individuals with a laboratory-confirmed positive result. Specifically, the number of ESBL-PE positive participants was divided by the total number of participants screened in that study, and the result was multiplied by 100 to yield a percentage.

### Data extraction and management

All retrieved records were imported into EndNote version 9.2 for reference management and duplicate removal. Following title and abstract screening, full-text articles were assessed for eligibility. Data extraction was conducted independently by two reviewers (AZ. And MG.) using a standardized Microsoft Excel 2019 form. Extracted variables included primary author, publication year, study design, study period and setting, geographical region, sample size, number of cases, ESBL-PE detection method, and the rates of overall ESBL-PE colonization as well as individual bacteria species. Any discrepancies in the extracted data were resolved through discussion until a consensus was reached.

### Data synthesis and statistical analysis

Statistical analyses were performed using Stata version 17 (StataCorp, 2021) [30]**.** Given the considerable heterogeneity across studies, pooled estimates were calculated using a random-effects approach based on the DerSimonian–Laird method [31]. The Wilson score method was used to calculate 95% confidence intervals for proportions. This approach is especially recommended when proportions approach the boundaries of 0 or 1, or when the number of included studies is small, as it constrains the interval within the mathematically valid [0, 1] range.

The degree of statistical heterogeneity among the included studies was quantified using the I^2^ statistic. The I^2^ values were interpreted as follows: less than 25% represented low heterogeneity, 25% to 50% indicated moderate heterogeneity, 50% to 75% represented substantial heterogeneity, and values exceeding 75% signified considerable heterogeneity. The statistical significance of the observed heterogeneity was determined by the *p* value of the Cochrane Q statistic, and *p*-value of less than 0.05 was evidence of heterogeneity [32].

### Subgroup and sensitivity analysis

Given the significant statistical heterogeneity identified among the included studies, we conducted in-depth analysis to explore its potential sources and assess the robustness of our findings. A subgroup analysis was performed by stratifying the studies based on three pre-specified factors: the geographic region in Africa (Eastern, Western, and Northern,), study year (2012–2019, and 2020–2025), and the specific laboratory method used for ESBL-PE detection double disk synergy test (DDST), and combination disk test (CDT). Additionally, a leave-one-out sensitivity analysis was conducted to assess the influence of individual studies on the overall pooled prevalence of ESBL-PE [31].

### Publication bias

The potential for publication bias and small study effects across the included studies was assessed using both graphical and statistical methods. A funnel plot was generated and visually inspected for symmetry; a symmetrical, inverted funnel shape suggested a low likelihood of publication bias. To complement this visual assessment, Egger’s regression test was employed. A *p*-value greater than 0.05 from Egger’s test was interpreted as indicating no statistically significant publication bias [33].

### Ethics approval and consent to participate

Since this study was based on data extracted from previously published studies, ethical approval was not applicable.

## Results

### Selection of studies

The systematic search across scientific databases initially yielded 752 records. Following the removal of 37 duplicates, 715 unique articles remained for screening. After a review of the titles and abstracts, 672 records were excluded as irrelevant. The full texts of the remaining 43 articles were then assessed for eligibility based on the predefined inclusion criteria and quality appraisal. Finally, 9 studies were eligible and included in the final meta-analysis, as detailed in the PRISMA flow diagram (Fig 1).

### Summary of quality appraisal of included studies

Regarding the studies that met the quality criteria, 5 out of 9 (55.6%) had a good quality, while 4 (44.4%) had a moderate quality. No studies were excluded due to a low quality **(Table 1).**

**Table 1:** Quality appraisal of included studies evaluating the fecal colonization rate of ESBL-PE among Food handlers in Africa, 2025.

Author, Year	Study design	Q1	Q2	Q3	Q4	Q5	Q6	Q7	Q8	Q9	Score out of 9	Remark
Ahmed et al, 2023 [44]	CS	UN	No	No	Yes	Yes	Yes	Yes	Yes	Yes	6	Included
Amare et al, 2022 [45]	CS	Yes	Yes	Yes	Yes	Yes	Yes	Yes	Yes	Yes	9	Included
Aworh et al, 2020 [46]	CS	No	Yes	No	Yes	Yes	Yes	Yes	Yes	Yes	7	Included
Diriba et al, 2020 [47]	CS	Yes	Yes	UN	No	Yes	Yes	Yes	Yes	Yes	7	Included
Godonou et al, 2019 [36]	CS	UN	Yes	No	Yes	Yes	Yes	Yes	Yes	Yes	7	Included
Juma et al, 2017 [34]	CS	Yes	Yes	Yes	Yes	Yes	Yes	Yes	Yes	Yes	9	Included
Mwanginde et al, 2021 [48]	CS	Yes	Yes	Yes	Yes	Yes	Yes	Yes	Yes	Yes	9	Included
Sallem et al, 2012–2017 [49]	CS	Yes	No	Yes	Yes	Yes	Yes	Yes	Yes	Yes	8	Included
Sanneh et al, 2015 [35]	CS	Yes	Yes	Yes	Yes	Yes	Yes	Yes	Yes	Yes	9	Included

**Abbreviations:** CS = Cross-sectional; UN = unclear; Q = Question.

**Note:** The overall score is calculated by counting the number of Yes’s in each row.

The following quality appraisal questions were for cross-sectional studies: Q1: Was the sample frame appropriate to address the target population? Q2: Were study participants sampled in an appropriate way? Q3: Was the sample size adequate? Q4: Were the study subjects and the setting described in detail? Q5: Was the data analysis conducted with sufficient coverage of the identified sample? Q6: Were valid methods used for the identification of the condition? Q7: Was the condition measured in a standard reliable way for all participants? Q8: Was there appropriate statistical analysis? And Q9: Was the response rate adequate, and if not, was the low response rate managed appropriately were for cross-sectional studies.

### Characteristics of the studies included in the systematic review and meta-analysis

This systematic review and meta-analysis incorporated nine cross-sectional studies published between 2012 and 2023, encompassing a total of 4,061 participants. The sample sizes of the individual studies varied considerably, ranging from 50 to 2,135. Regarding ESBL-PE detection methods, the majority of studies (seven) utilized the DDST, while the remaining two employed the CDT. Geographically, the studies were distributed as follows: four from Eastern Africa, three from Western Africa, and two from Northern Africa. In terms of reported outcomes, seven studies specifically documented ESBL-producing *E. coli*, while only four studies reported ESBL-producing *Klebsiella* species. Additionally, only two studies were reported ESBL-PE other than *E. coli* and *Klebsiella* species (*Proteus* species, *Citrobacter* species, and *Enterobacter* specie). Furthermore, multidrug resistance (MDR) among ESBL-PE isolates was clearly detailed in only three studies. This consistent finding of 100% MDR across all three studies may be explained by the fact that ESBL-PE frequently co-harbor resistance genes to multiple antibiotic classes, as ESBL genes are often located on mobile genetic elements (plasmids) that carry additional resistance determinants (**Table 2 and** **S1 Data**).

**Table 2:** Characteristics of studies included in the meta-analysis of ESBL-PE colonization among food handlers in Africa, 2025.

Study (Author, year)	Study year	Study area	Study region	Study population (Setting)	Sample size	ESBL-Method	No of cases	Total ESBL-PE (%)	ESBL-*E. coli* N (%)	ESBL-*Klebsiella* species N (%)	ESBL-others N (%)	ESBL-MDR N (%)
Ahmed et al, 2023 [44]	2023	Egypt	Northern Africa	Retail meat shop workers	50	DDST	11	22%	11 (100.0%)	NR	NR	11 (100.0%)
Amare et al, 2022 [45]	2022	Ethiopia	Eastern Africa	University cafeterias	290	CDT	63	21.7%	43 (68.0%)	17 (27.0%)	3 (4.8%)	63 (100.0%)
Aworh et al, 2020 [46]	2020	Nigeria	Western Africa	Slaughterhouse workers	118	CDT	19	16.1%	19 (100.0%)	0	0	NS
Diriba et al, 2020 [47]	2020	Ethiopia	Eastern Africa	University cafeterias	220	DDST	37	16.8%	29 (78.4%)	8 (21.6%)	NR	NS
Godonou et al, 2019 [36]	2019	Togo	Western Africa	Slaughterhouse workers	60	DDST	48	80.0%	NS	NS	NS	NR
Juma et al, 2017 [34]	2017	Kenya	Eastern Africa	Hotels	323	DDST	11	3.4%	11 (100.0%)	0	0	11 (100.0%)
Mwanginde et al, 2021 [48]	2021	Tanzania	Eastern Africa	Poultry meat vendors	300	DDST	107	35.7%	84 (78.5%)	23 (21.5%)	0	NS
Sallem et al, 2012–2017 [49]	2012-2017	Tunisia	Northern Africa	City food handlers	2135	DDST	419	19.6%	NS	NS	NS	NS
Sanneh et al, 2015 [35]	2015	Gambia	Western Africa	lower basic schools	565	DDST	28	5.0%	8 (28.6%)	9 (32.1%)	5 (17.9%)	NS

**Abbreviations:** CS = Cross-sectional study; CDT = Combination disk test; DDST = Double disk synergy test; NR = Not-reported; NS = Not specified; ESBL-PE = Extended-spectrum Beta-lactamase-producing Enterobacteriaceae; ESBL-PE Methods = Methods of confirmation for ESBL-PE; ESBL others: *Proteus* species, *Citrobacter* species, and *Enterobacter* species.

### Pooled Colonization rate of ESBL-PE among food handlers in Africa

**Fig 2:**
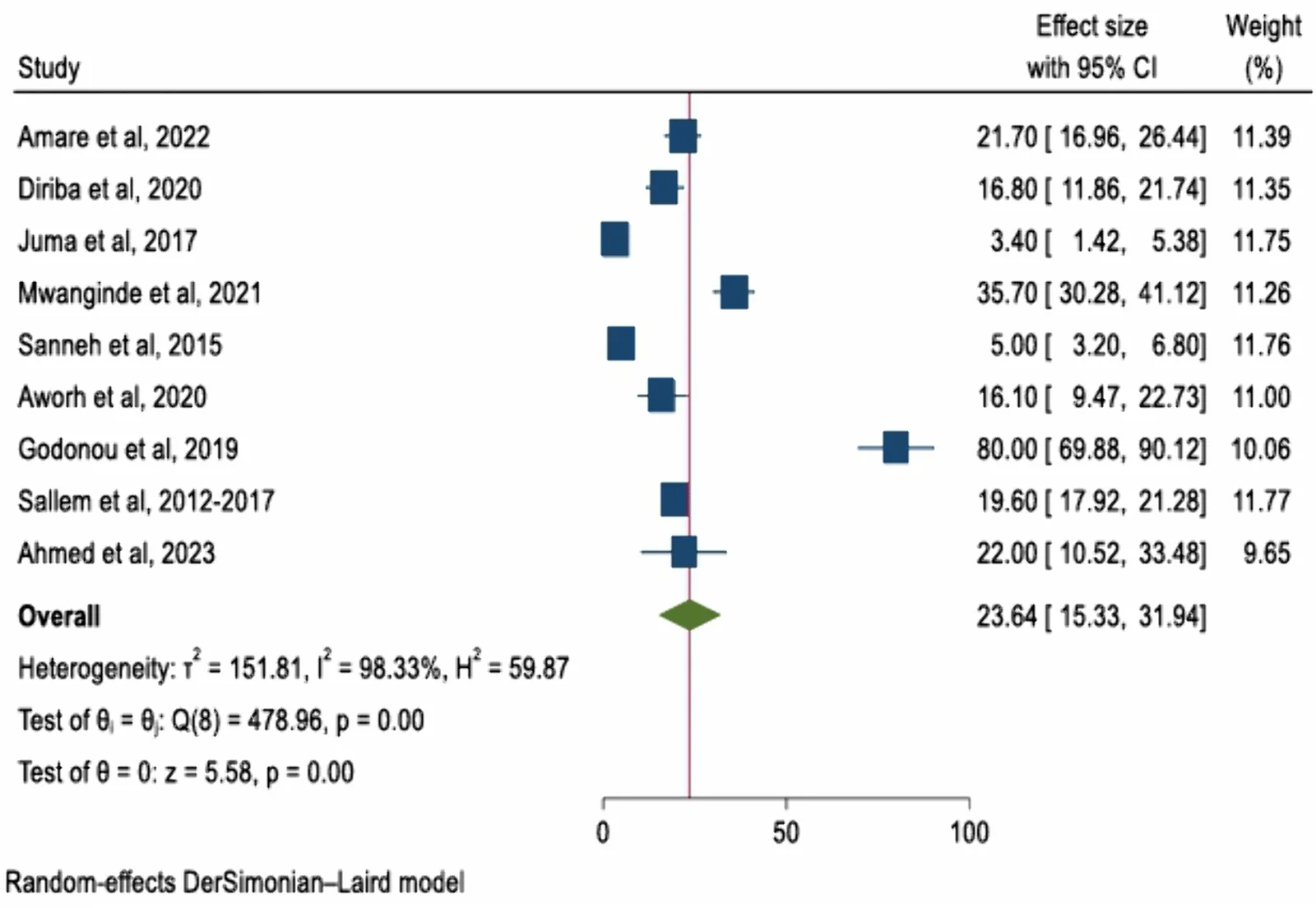
Forest plot showing the pooled prevalence of fecal colonization with ESBL-producing Enterobacteriaceae among food handlers in Africa using a random-effects model.

The meta-analysis on the fecal colonization of ESBL-PE among food handlers in Africa gives a pooled prevalence of 23.64%. The nine included studies reported a prevalence ranging from 3.4% to 80.0% (Fig 2).

**Fig 3:**
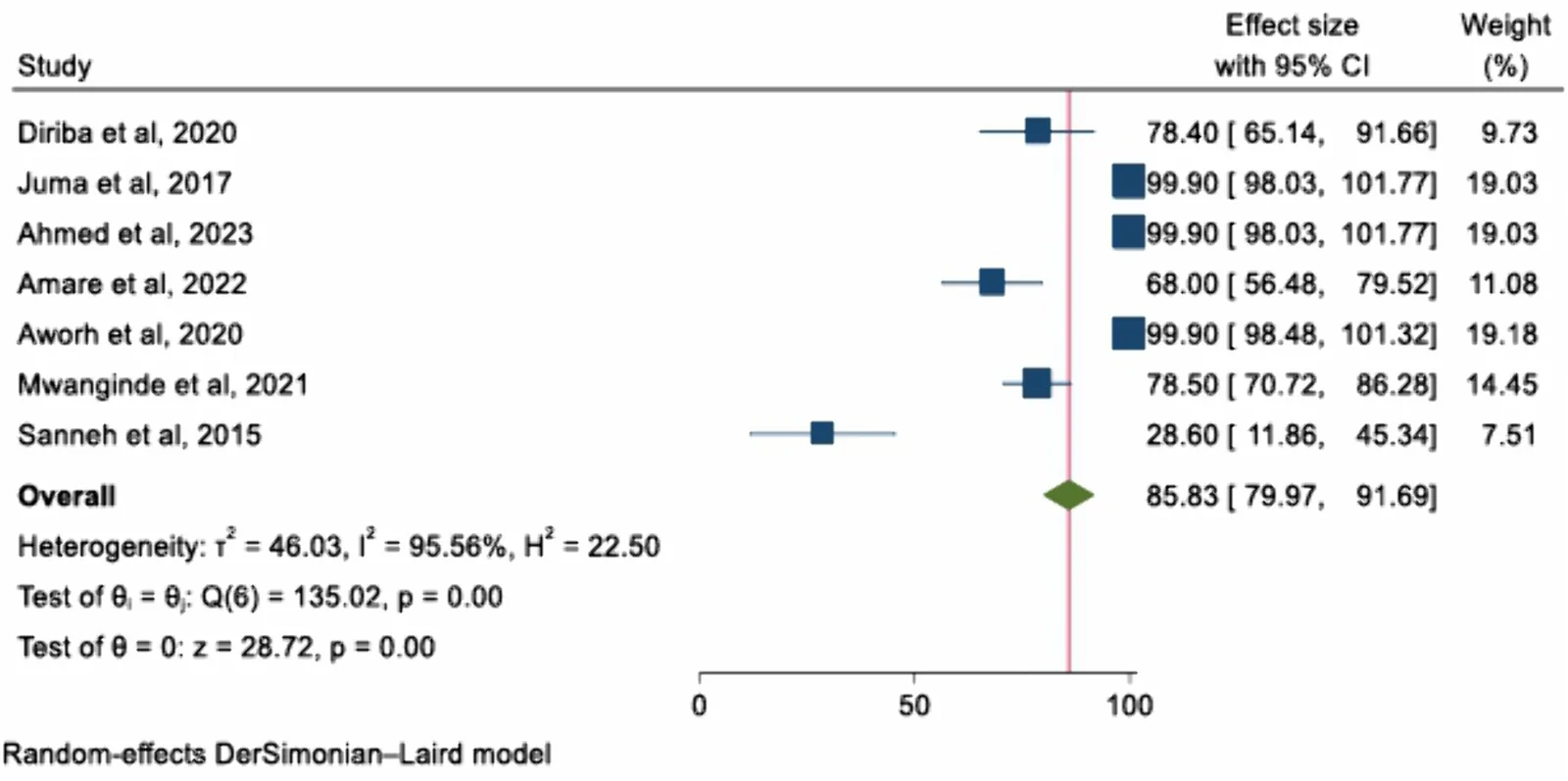
Forest plot showing the pooled prevalence of fecal colonization with ESBL-producing *E. coli* among food handlers in Africa using a random-effects model.

Furthermore, the meta-analysis on the fecal colonization of ESBL-producing *E. coli* among food handlers in Africa gives a pooled prevalence of 85.83%. Only seven studies were reported ESBL-producing *E. coli* **(**Fig 3).

**Fig 4:**
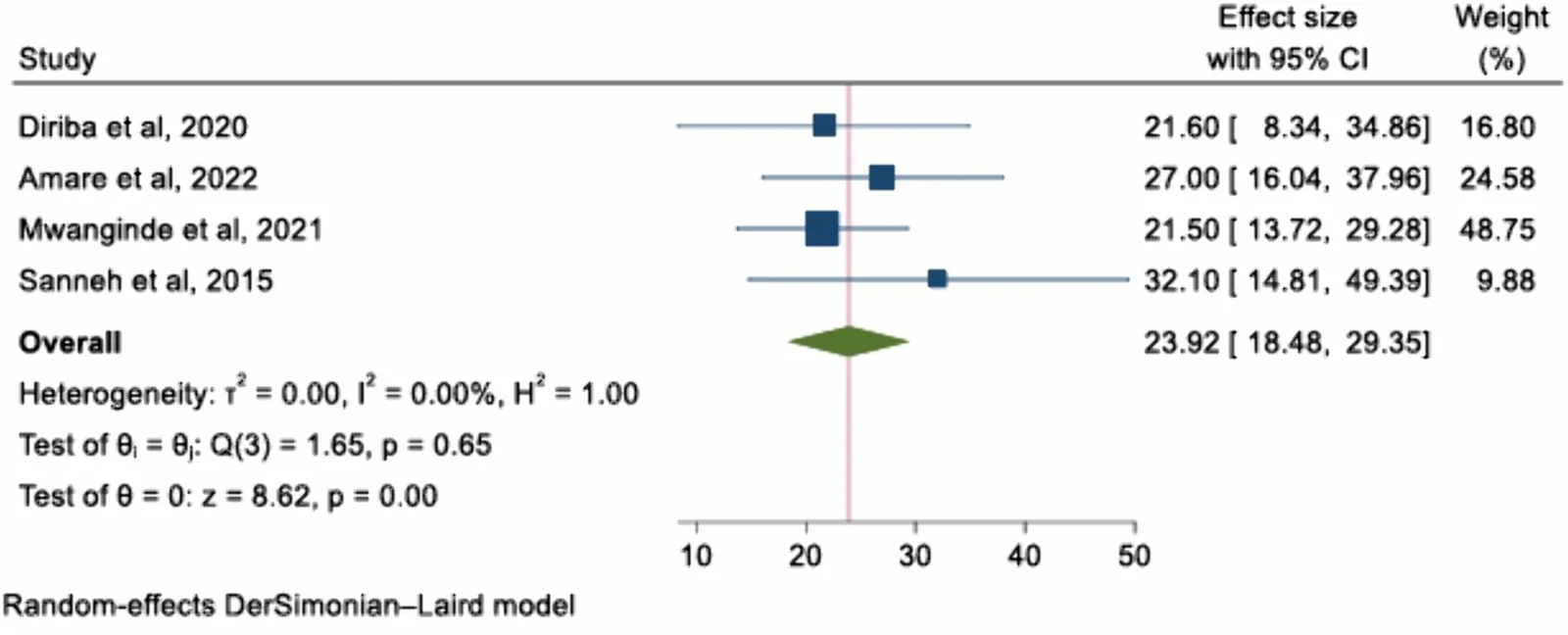
Forest plot showing the pooled prevalence of fecal colonization with ESBL-producing *Klebsiella* species among food handlers in Africa using a random-effects model.

Additionally, the meta-analysis on the fecal colonization of ESBL- producing *Klebsiella* species among food handlers in Africa gives a pooled prevalence of 23.92%. Only four studies were reported ESBL-producing *Klebsiella* species (Fig 4).

The forest plot visually represents these findings. Each study is depicted by a blue square, indicating its individual point estimate (colonization rate), and a horizontal line, representing its 95% confidence interval. The width of these lines reflects the precision of each estimate, with wider lines indicating greater uncertainty. A diamond at the bottom summarizes the overall pooled estimate, its center marks the point prevalence, while its width shows the 95% CI for the combined result (Figs 2-4).

According to this meta-analysis, the pooled colonization rate of ESBL-PE among food handlers in Africa was 23.64% (95% CI: 15.3, 31.94%). A very high degree of statistical heterogeneity was observed among the study results (I^2^ = 98.3%, *p* < 0.001) (Fig 2), suggesting that the true prevalence varies considerably across the included studies. Therefore, a random effects model was used to estimate the average effect size (prevalence). Furthermore, to explore the potential sources of this heterogeneity, subgroup and sensitivity analyses were performed **Table 3**.

**Table 3:** Subgroup analysis of ESBL-PE among Food handlers in Africa, 2025.

Characteristics	No of studies	Sample size	pooled colonization rate of ESBL-PE (95% CI)	Heterogeneity	
				I^2^ (%)	*P* –value
**Regions**					
Eastern Africa	4	1133	19.28% (95% CI: 4.67, 33.89)	98.12	<0.001
Western Africa	3	743	33.3% (95% CI: −0.93, 67.6)	99.05	<0.001
Northern Africa	2	2185	19.65% (95% CI: 17.98, 21.32)	0.00	0.69
**ESBL detection methods**					
DDST	7	3653	25.06% (95% CI: 15.29, 34.84)	44.81	0.18
CDT	2	408	19.40% (95% CI: 14.00, 24.80)	98.70	<0.001
**Study year**					
2012-2019	4	3083	25.08% (95% CI: 12.52, 37.64)	99.21	<0.001
2020-2025	5	978	22.53% (95% CI: 14.94, 30.11)	87.37	<0.001

**Abbreviations:** CI = Confidence Interval; CDT = Combination disk test; DDST = Double disk synergy test; ESBL-PE = Extended-spectrum Beta-lactamase-producing Enterobacteriaceae.

Significant variation was evident across the individual studies. The lowest colonization rates were reported in Kenya (3.4%) [34] and Gambia (5%) [35], while the highest rate was found in a study from Togo (80%) [36].

The most prevalent ESBL-PE species identified were *E. coli*, with a pooled prevalence of 85.83% (95% CI: 79.97, 91.69%, I^2^ = 95.56%, *p* < 0.001) (Fig 3), followed by *Klebsiella* species at 23.92% (95% CI: 18.48, 29.35%, I^2^ = 0.00%, *p* = 0.65) **(**Fig 4**)**, and ESBL-PE other than *E. coli* and *Klebsiella* species at 6.0% (95% CI: 1.0, 10.0%, I^2^ = 0.00%, *p* < 0.001) (**S1 Fig****)***.* In terms of MDR, only three studies reported it among ESBL-PE with 100%.

### Subgroup analysis of the ESBL-PE colonization rate

In an effort to explain the substantial heterogeneity observed across the studies, we performed a subgroup analysis based on three key variables: geographic region, diagnostic method for ESBL-PE confirmation, and the study period. The analysis revealed notable regional differences. The highest pooled colonization rate of ESBL-PE was in Western Africa at 33.3% (95% CI: −0.93, 67.6), followed by Northern Africa at 19.65% (95% CI: 17.98, 21.32), and eastern Africa at 19.28% (95% CI: 4.67, 33.89). Although the Western Africa subgroup showed the highest point estimate (33.3%), its 95% CI was wide and crossed zero, indicating that this finding is statistically imprecise and should be interpreted with caution. When stratified by detection method used for ESBL production, studies using the DDST reported a higher pooled prevalence of 25.06% (95% CI: 15.29, 34.84) compared to the 19.40% (95% CI: 14.00, 24.80) found in studies using the CDT. Subgroup analysis in terms of study year showed a slightly higher prevalence in studies conducted between 2012 and 2019, at 25.08% (95% CI: 12.52, 37.64), compared to those from 2020 to 2025, which had a prevalence of 22.53% (95% CI: 14.94, 30.11), as indicated in **(Table 3 and** **S2 Figs****).**

### Publication bias

**Fig 5:**
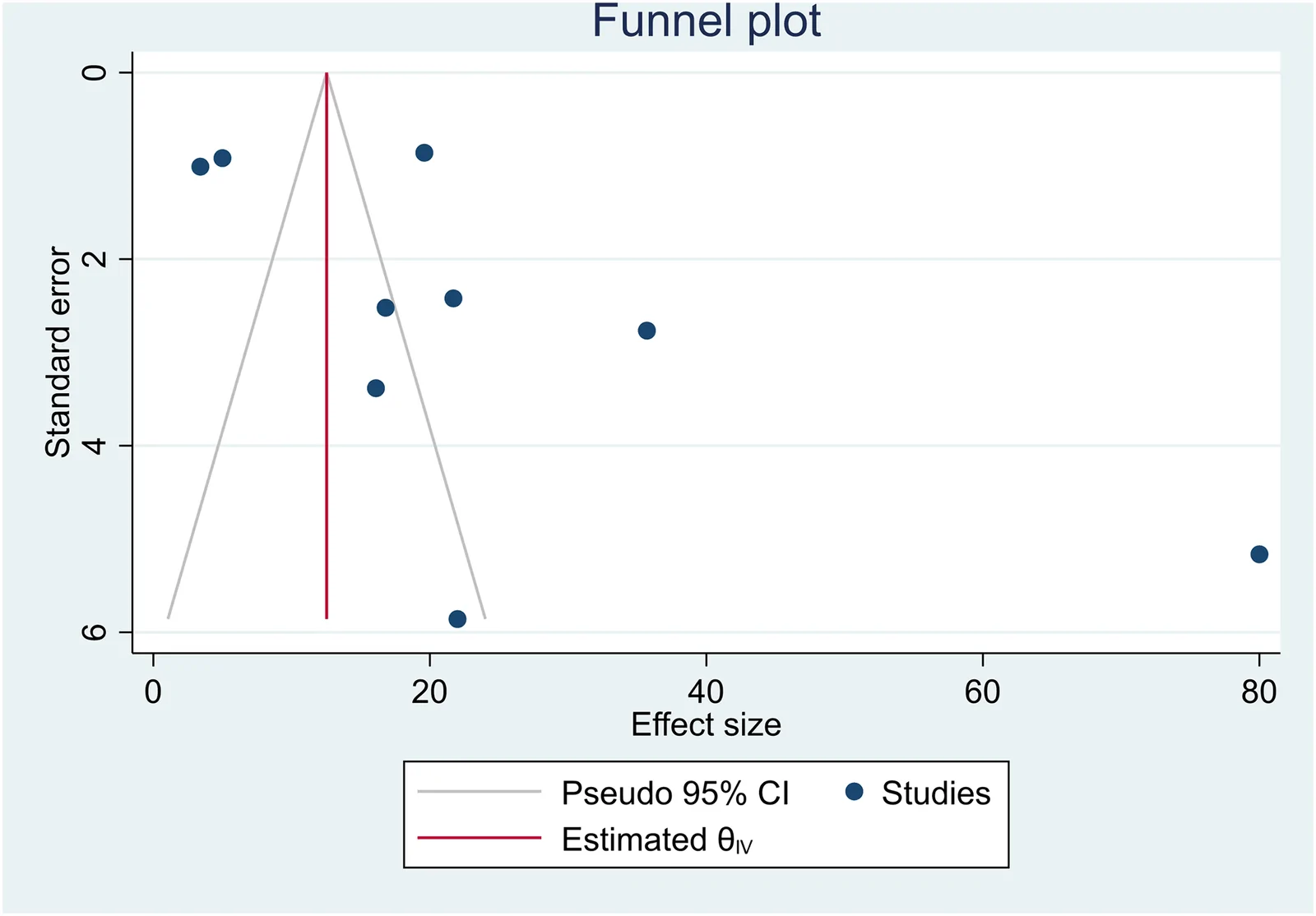
Funnel plot assessing potential publication bias among studies reporting ESBL-PE colonization in food handlers.

Publication bias was assessed to determine whether the selected studies represented the original population or were influenced by bias related to published and unpublished studies. The ESBL-PE analysis revealed an asymmetric funnel plot, indicating the presence of publication bias (Fig 5).

Similarly, Egger’s test showed significant publication bias (*p* = 0.003) (**Table 4).**

**Table 4:** Egger’s test for the pooled estimates of the colonization rates of ESBL-PE among Food handlers in Africa, 2025.

**ESBL-PE**	**N**o **of studies**	**pooled colonization rate of ESBL-PE (95% CI)**	**Heterogeneity**		**Eggers regression test**	
			**I**^**2**^ **(%)**	***P* -value**	***P* -value**	**95% CI**
	9	23.64% (95% CI: 15.3, 31.94%)	98.3%	<0.001	0.003	3.67, 12.42

**Abbreviations:** CI = Confidence Interval; ESBL-PE = Extended-spectrum Beta-lactamase-producing Enterobacteriaceae.

### Trim-and-fill analysis of the pooled colonization rate of ESBL-PE

To address publication bias, a trim-and-fill analysis was performed. Without imputing data on the left, the analysis found that the pooled colonization rate of ESBL-PE among food handlers in Africa remained stable at 23.64% (95% CI: 15.3, 31.94%) (**Table 5 and** **S3 Fig****).** Conversely, when analyzing 12 studies with three data points imputed on the right, the pooled colonization rate was slightly higher at 32.23% (95% CI: 17.31, 47.14%) (**Table 6 and** **S3 Fig**). This upward adjustment indicates that smaller studies with higher ESBL‑PE prevalence are likely missing from the published literature.

**Table 5:** Nonparametric trim-and-fill analysis of publication bias imputing on the left.

Nonparametric trim-and-fill analysis of publication bias	
**Linear estimator, imputing on the left**	
**Iteration**	
Parameter	Value
No of studies	9
Model	Random-effects
Method	DerSimonian-Laird
Observed	9
Imputed	0
**Pooling**	
Parameter	Value
Model	Random-effects
Method	DerSimonian-Laird
**Results**	
Studies	Effect size (95% CI)
Observed	23.637% (95% CI: 15.331, 31.943%)
Observed + Imputed	23.637% (95% CI: 15.331, 31.943%)

**Abbreviations:** CI = Confidence Interval.

**Table 6:** Nonparametric trim-and-fill analysis of publication bias imputing on the right.

Nonparametric trim-and-fill analysis of publication bias	
**Linear estimator, imputing on the right**	
**Iteration**	
Parameter	Value
No of studies	12
Model	Random-effects
Method	DerSimonian-Laird
Observed	9
Imputed	3
**Pooling**	
Parameter	Value
Model	Random-effects
Method	DerSimonian-Laird
**Results**	
Studies	Effect size (95% CI)
Observed	23.637% (95% CI: 15.331, 31.943%)
Observed + Imputed	32.226% (95% CI: 17.312, 47.139%)

**Abbreviations:** CI = Confidence Interval.

### Sensitivity analysis of ESBL-PE colonization rate

Sensitivity analysis was carried out using leave-one-out approach to detect any potential outlier studies. Based on the random effects model, no single study had a disproportionate impact on the overall pooled estimates of ESBL-PE colonization rates. The findings revealed that the estimates from all included studies were within the pooled estimate’s confidence interval, indicating the reliability of the aggregated results (**Table 7 and** **S4 Fig**).

**Table 7:** Sensitivity analysis of ESBL-PE colonization rate among Food handlers in Africa, 2025.

Sensitivity analysis of ESBL-PE			
Study omitted	Estimate	95% CI	*P*-value
Ahmed et al, 2023 [44]	23.82	15.05, 32.59	<0.001
Amare et al, 2022 [45]	23.92	14.93, 32.91	<0.001
Aworh et al, 2020 [46]	24.60	15.65, 33.55	<0.001
Diriba et al, 2020 [47]	24.56	15.49, 33.63	<0.001
Godonou et al, 2019 [36]	17.23	10.11, 24.36	<0.001
Juma et al, 2017 [34]	26.42	17.01, 35.83	<0.001
Mwanginde et al, 2021 [48]	22.01	13.64, 30.37	<0.001
Sallem et al, 2012–2017 [49]	24.36	14.41, 34.32	<0.001
Sanneh et al, 2015 [35]	26.27	16.42, 36.13	<0.001
**Pooled**	**23.64**	**15.3, 31.94**	

**Abbreviations:** CI = Confidence Interval = ESBL-PE = Extended-spectrum Beta-lactamase-producing Enterobacteriaceae.

## Discussion

This systematic review and meta-analysis provides a comprehensive estimate of fecal colonization with extended-spectrum β-lactamase producing Enterobacteriaceae (ESBL-PE) among food handlers in Africa. The pooled prevalence indicates that nearly one-quarter of food handlers carry ESBL-PE in their intestinal tract, highlighting a substantial reservoir of antimicrobial-resistant bacteria within the community. This finding underscores the public health importance of food handlers as a potential link between antimicrobial resistance in the environment, food systems, and the general population.

The observed prevalence is comparable to colonization rates reported among other high-risk populations, including residents of long-term care facilities (18%) [21], and patients with hematological malignancies (19%) [37], suggesting that occupational exposure may play an important role in ESBL-PE acquisition. In addition, the estimated prevalence aligns closely with prior reviews conducted in Ethiopia across mixed populations (28.5%) [24], indicating that ESBL-PE colonization is not confined to healthcare environments but is widely established in the community. Factors such as unregulated antibiotic use, limited sanitation infrastructure, and inadequate infection prevention practices may contribute to this widespread distribution.

When compared with hospital-based studies from Africa (32%) [22], and global analyses among hospitalized patients (45.6%) [20], the pooled prevalence in food handlers was lower. This difference may reflect the increased antibiotic exposure, invasive procedures, and prolonged healthcare contact experienced by hospitalized individuals, all of which are known risk factors for colonization with resistant organisms [38]. Conversely, the prevalence among food handlers was higher than estimates reported in general healthy community populations worldwide (14%) [39], supporting the notion that food handlers constitute a distinct subgroup with elevated exposure risk, potentially through contact with contaminated food products, raw animal materials, or suboptimal hygiene conditions [40].

Regarding to detected significant publication bias both visually (asymmetric funnel plot) and statistically (Egger’s test, p = 0.003). Trim‑and‑fill analysis imputing missing studies on the right raised the pooled estimate from 23.64% to 32.23%, suggesting that the true prevalence may be higher than our original estimate. However, this correction should be interpreted cautiously, as imputed studies are hypothetical. Potential sources of bias include small‑study effects, language bias, or underreporting of negative findings. Therefore, the headline pooled estimate of 23.64% may underestimate the true prevalence, and readers should consider both original and adjusted estimates

Subgroup analyses revealed considerable regional variation in ESBL-PE colonization across Africa. Studies conducted in Western Africa demonstrated the highest pooled prevalence (33.3%), which may be partly explained by the inclusion of slaughterhouse workers in this subgroup, who have frequent contact with live animals and raw meat, recognized reservoirs of ESBL-producing bacteria, may increase occupational exposure and colonization risk [41]. Another possible reason for the elevated ESBL‑PE carriage in this subgroup may be the inclusion of the Togo study (80% prevalence), in which the majority of slaughterhouse workers practiced antibiotic self‑medication, had ≥ 10 years of work experience, and used borehole water at their work site [8]. The apparently higher prevalence estimate for Western Africa (33.3%) was not statistically robust, as reflected by a confidence interval that included zero. This may be due to the small number of studies (n = 3) or high heterogeneity (99.05%) in that subgroup. In contrast, studies from Eastern Africa reported lower prevalence estimates (19.28%), possibly reflecting differences in food handling practices, and workplace hygiene standards.

A subgroup analysis suggested a modest reduction in ESBL-PE colonization in studies conducted after 2020. This trend may be influenced by enhanced hygiene practices implemented during the COVID-19 pandemic, including increased hand hygiene awareness, surface disinfection, and general infection prevention measures, which could have indirectly reduced the transmission of enteric bacteria [42].

Furthermore, variation in prevalence was also observed based on laboratory detection methods. Studies employing the DDST reported higher colonization rates compared with those using the CDT. This discrepancy likely reflects methodological differences in sensitivity, as DDST is more sensitive for detecting weak ESBL producers and a broader range of ESBL types, whereas CDT is generally regarded as more specific. However, DDST results are highly dependent on optimal disk spacing [43].

## Limitations

Despite its strengths, this review has limitations. The substantial heterogeneity observed across studies suggests variability in study design, population characteristics, laboratory methods, and regional contexts. Additionally, the majority of included studies were cross-sectional and originated from a limited number of African countries, which may restrict the generalizability of the findings. Furthermore, data on risk factors, were inconsistently reported, precluding more detailed analysis. Finally, the lack of molecular characterization of genes across the majority of these primary studies prevents pooling of ESBL genes.

## Conclusion and recommendations

This systematic review and meta-analysis demonstrates that fecal colonization with ESBL-PE is common among food handlers in Africa, indicating an underappreciated pathway for the dissemination of antimicrobial-resistant bacteria in the community. Given the central role of food handlers in food preparation and distribution, their intestinal carriage of ESBL-PE represents a potential risk for indirect transmission to consumers and the wider population.

To mitigate this threat, targeted public health interventions are warranted. These should include routine health screening and microbiological surveillance among food handlers, strengthened food safety regulations, and improved access to hand hygiene and sanitation facilities. In parallel, the implementation of robust antimicrobial stewardship programs is essential to reduce inappropriate antibiotic use in both community and occupational settings. Future research should prioritize longitudinal studies and molecular investigations to better understand transmission dynamics and inform effective prevention strategies.

## Supporting information

S1 Table PRISMA checklist, 2020. (DOCX)

S2 Table PubMed Search strategy. (DOCX)

S1 Fig Forest plot of ESBL-PE other than E.coli and Klebsiella species. (DOCX)

S2 Fig Forest plot of subgroup analysis. (DOCX)

S3 Fig Forest plot of sensitivity analysis. (DOCX)

S4 Figs Funnel plot of trim and fill analysis. (DOCX)

S1 Data Raw data of our meta-analysis. (XLSX)

## Abbreviations

AMR: Antimicrobial Resistance
CDC: Centers for Disease Control and Prevention
CDT: Combination Disk Test
CI: Confidence Interval
CoCoPop: Condition, Context, and Population
DDST: Double Disk Synergy Test
ESBL: Extended Spectrum Beta-Lactamase
ESBL-PE: Extended Spectrum Beta-Lactamase-Producing Enterobacteriaceae
JBI: Joanna Briggs Institute
MDR: Multidrug Resistance
MeSH: Medical Subject Heading
PRISMA: Preferred Reporting Items for Systematic Reviews and Meta-Analysis
US: United States
WHO: World Health Organization
