# Supplementary material for: Burden of fecal colonization with extended-spectrum beta-lactamase producing enterobacteriaceae among food handlers in Africa: A systematic review and meta-analysis
Source: PLoS One. 2026 Aug 24;21(8):e0340571. doi: 10.1371/journal.pone.0340571 (PMC13502540; doi:10.1371/journal.pone.0340571)
Supplement: Supplementary file 2 [file pone.0340571.s002.docx]

**S2 Table:** Search strategy for PubMed

| **1** | (("Fecal carriage" OR "Fecal colonization" OR "Gut carriage" OR "Gut colonization" OR "Intestinal colonization" OR "Intestinal carriage" OR Colonization OR Carriage)) |
| --- | --- |
| **2** | (("Extended spectrum beta lactamase" OR "Extended spectrum β lactamase" OR ESBL OR ESβL OR Beta-Lactamase)) |
| **3** | ((Enterobacteriaceae OR Enterobacterales OR "Gram-Negative Bacteria" OR "Gram-Negative Bacteria"[Mesh] OR Enterobacteriaceae[Mesh] OR Klebsiella OR Klebsiella[Mesh] OR "Escherichia coli" OR "Escherichia coli"[Mesh] OR E.coli OR Shigella OR Shigella[Mesh] OR Salmonella OR Salmonella[Mesh])) |
| **4** | ((Africa OR Africa[Mesh] OR "Sub Saharan Africa" OR "Africa South of the Sahara"[Mesh])) |
| **5 (1 AND 2):** | (("Fecal carriage" OR "Fecal colonization" OR "Gut carriage" OR "Gut colonization" OR "Intestinal colonization" OR "Intestinal carriage" OR Colonization OR Carriage)) AND (("Extended spectrum beta lactamase" OR "Extended spectrum β lactamase" OR ESBL OR ESβL OR Beta-Lactamase)) |
| **6 (3 AND 4)** | ((Enterobacteriaceae OR Enterobacterales OR "Gram-Negative Bacteria" OR "Gram-Negative Bacteria"[Mesh] OR Enterobacteriaceae[Mesh] OR Klebsiella OR Klebsiella[Mesh] OR "Escherichia coli" OR "Escherichia coli"[Mesh] OR E.coli OR Shigella OR Shigella[Mesh] OR Salmonella OR Salmonella[Mesh])) AND ((Africa OR Africa[Mesh] OR "Sub Saharan Africa" OR "Africa South of the Sahara"[Mesh])) |
| **7 (5 AND 6)** | (("Fecal carriage" OR "Fecal colonization" OR "Gut carriage" OR "Gut colonization" OR "Intestinal colonization" OR "Intestinal carriage" OR Colonization OR Carriage)) AND (("Extended spectrum beta lactamase" OR "Extended spectrum β lactamase" OR ESBL OR ESβL OR Beta-Lactamase)) AND ((Enterobacteriaceae OR Enterobacterales OR "Gram-Negative Bacteria" OR "Gram-Negative Bacteria"[Mesh] OR Enterobacteriaceae[Mesh] OR Klebsiella OR Klebsiella[Mesh] OR "Escherichia coli" OR "Escherichia coli"[Mesh] OR E.coli OR Shigella OR Shigella[Mesh] OR Salmonella OR Salmonella[Mesh])) AND ((Africa OR Africa[Mesh] OR "Sub Saharan Africa" OR "Africa South of the Sahara"[Mesh])) |
