# Supplementary material for: Burden of fecal colonization with extended-spectrum beta-lactamase producing enterobacteriaceae among food handlers in Africa: A systematic review and meta-analysis
Source: PLoS One. 2026 Aug 24;21(8):e0340571. doi: 10.1371/journal.pone.0340571 (PMC13502540; doi:10.1371/journal.pone.0340571)
Supplement: Supplementary file 5 [file pone.0340571.s005.docx]

**S3 Fig**

**Funnel plot of the nonparametric trim-and-fill analysis of publication bias imputing studies on the right**

**Funnel plot of the nonparametric trim-and-fill analysis of publication bias without imputing studies on the left**
