# Supplementary material for: Burden of fecal colonization with extended-spectrum beta-lactamase producing enterobacteriaceae among food handlers in Africa: A systematic review and meta-analysis
Source: PLoS One. 2026 Aug 24;21(8):e0340571. doi: 10.1371/journal.pone.0340571 (PMC13502540; doi:10.1371/journal.pone.0340571)
Supplement: Supplementary file 6 [file pone.0340571.s006.docx]

**S4 Fig**

**Forest plot of the sensitivity analysis using the leave-one-out approach of the colonization rates of ESBL-PE among food handlers in Africa, 2025**
